# Mid-Term Results of Fenestrated Endovascular Repair after Prior Open Aortic Reconstruction

**DOI:** 10.3390/jcm11195596

**Published:** 2022-09-23

**Authors:** Pablo Marques de Marino, Melad Abu Jiries, Pavel Tesinsky, Anas Ibraheem, Athanasios Katsargyris, Eric L. Verhoeven

**Affiliations:** Department of Vascular and Endovascular Surgery, General Hospital Nuremberg, Paracelsus Medical University, 90471 Nuremberg, Germany

**Keywords:** endovascular procedures, fenestrated endovascular aneurysm repair, abdominal aortic aneurysm, juxtarenal aortic aneurysm, previous aortic repair, para-anastomotic aneurysm

## Abstract

This study aims to assess the mid-term results of fenestrated endovascular aneurysm repair (FEVAR) for the treatment of proximal aortic pathology after previous open surgical repair (OSR). All patients with a previous history of OSR of an abdominal aortic aneurysm undergoing a FEVAR procedure between October 2010 and November 2021 were included. The endpoints of the study were technical success, mortality, target vessel patency and reinterventions during follow-up. Thirty-five patients (34 male, mean age 72.9 ± 7 years) were included. The median interval from the primary surgery to the FEVAR procedure was 136 months (range 47–261). The indication for treatment was a para-anastomotic aneurysm in 18 (51%) patients and a true aneurysm due to progression of disease in 17 (49%) patients. Technical success was achieved in 33 (94%) patients. There was one (3%) early death due to postoperative bleeding from a renal artery. Estimated survival at 12, 24 and 36 months was 89.1% ± 6%, 84.4% ± 7.3% and 84.4% ± 7.3%, respectively. There was no aneurysm-related mortality. One (3%) target vessel occluded during follow-up and three (9%) patients underwent late reinterventions. In conclusion, FEVAR is a safe and effective alternative for the endovascular treatment of para-anastomotic aneurysms/pseudoaneurysms after OSR showing high technical success, low mortality and morbidity, and good mid-term outcomes.

## 1. Introduction

Open surgical repair (OSR) has been considered the first treatment option for abdominal aortic aneurysms (AAA) for many years. OSR is associated with lower reintervention rates than endovascular aneurysm repair (EVAR) [1,2]. Still, the chronic nature of aneurysmal disease can contribute to the appearance of late aortic complications such as para-anastomotic aneurysms (PAA) and progression of disease with dilatation of the proximal aorta. Both clinical situations are challenging as they frequently involve the pararenal/paravisceral aorta and often occur in patients that are older and present more comorbidities than those treated for a primary AAA [3,4,5].

Open reintervention of this clinical scenario is associated with high mortality and morbidity rates [6,7,8] due to the redo nature of the surgery and the frequent need for suprarenal or supraceliac clamping.

The growing experience with fenestrated endovascular aortic aneurysm repair (FEVAR) and the good results reported with these techniques in juxta- and pararenal aneurysms [9,10,11] has opened a new option for the treatment of patients with proximal para-anastomotic aneurysms or extension of disease after previous open repair in centres with extensive experience in complex aortic endovascular repair.

The aim of this study is to assess the mid-term results of FEVAR for the treatment of proximal aortic pathology after previous open aortic reconstruction in our centre.

## 2. Methods

A retrospective single-centre study was conducted including all consecutive patients with previous history of OSR for AAA that underwent a FEVAR procedure between October 2010 and November 2021 to treat a para-anastomotic pseudoaneurysm (PAP) or a true aneurysm developing adjacent to the anastomosis. Data used for the analysis were taken from a prospectively maintained database. All patients signed informed consent for collection, processing and review of clinical and morphological data. Ethical committee approval was waived in view of the design of the study with anonymised retrospective data analysis.

### 2.1. Patients and Stent Graft Design

During the study period, all patients presenting in our centre with a PAP/PAA after OSR that needed repair were treated by endovascular means with a FEVAR procedure. All included patients presented an infrarenal landing zone shorter than 10 mm, which precluded a standard EVAR procedure. Patients with thoracoabdominal aortic aneurysms (TAAAs) that extended to the mid/proximal descending thoracic aorta were excluded from the study, as the nature of the disease and the extension of the treatment is different to those with PAP/PAAs. The fenestrated device was a custom-made stent graft based on the Zenith system (Cook Zenith CMD, Cook Medical Inc., Bloomington, IN, USA) in all cases. In patients with a distance < 35 mm from the lowest renal artery to the surgical graft bifurcation or the iliac bifurcation, a fenestrated cuff sealing in the surgical graft was planned. All other cases with sufficient working length were treated with a fenestrated tube graft followed by a bifurcated stent graft. This bifurcated device was designed to include an inverted contralateral limb in cases with a distance to the surgical graft bifurcation between 35 and 50 mm. The stent graft design and planning were completed by the senior author (E.L.V) based on the anatomical characteristics of the aneurysm, target vessels and previous surgical graft assessed by thin cut (≤1.5 mm) spiral computed tomography angiography (CTA) with axial, coronal and sagittal reconstructions. Indications for treatment and stent graft design in our centre have been previously described [12,13,14,15]. The physical status of all patients was assessed preoperatively with the American Society of Anesthesiologists (ASA) Physical Status Classification score.

### 2.2. Procedure

All procedures were performed under general anaesthesia in a hybrid operating room with a fixed imaging system and computed tomography fusion (Artis Zeego, Siemens Healthineers AG, Erlangen, Germany). Surgical access was performed with a bilateral femoral cutdown and double purse string sutures of Prolene (Ethicon, Somerville, NJ, USA) fitted with a snugger were used in all access vessels to potentially allow removal of the sheaths in order to restore blood flow to the artery. The detailed explanation of the FEVAR procedure execution in our centre has been previously described [15,16].

### 2.3. Follow-Up

Patients were prescribed dual antiplatelet treatment with aspirin and clopidogrel for at least 3 months and single antiplatelet treatment with aspirin thereafter unless the patient had a previous indication for anticoagulation in which the anticoagulant was combined with aspirin only. Surveillance with CTA imaging of the thoracoabdominal aorta was performed at 1 month, 1 year, and thereafter depending on each patient’s characteristics after discussion in the department. Upon suspicion of bridging stent stenosis/occlusion, endoleak or target vessel malperfusion, an additional angiography for evaluation and potential planning of a reintervention was carried out.

### 2.4. Endpoints and Statistical Analysis

Endpoints of the study were technical success, mortality, target vessel patency and reinterventions during follow-up. Technical success was defined as successful endovascular implantation of the stent graft with preservation of antegrade flow to the target vessels, and absence of type I or III endoleak at the first post-operative CTA.

Continuous data were presented as mean ± standard deviation in normally distributed variables and median and range if non-normally distributed. Baseline and comorbidity characteristics as well as intraoperative data were analysed. Qualitative variables were presented as total number and percentage. Patient survival, target vessel patency, and reinterventions during follow-up were subjected to Kaplan–Meier life table analysis. Data processing and analysis were performed using the SPSS^®^ statistical package for Windows, version 20.0 (SPSS, Chicago, IL, USA).

## 3. Results

### 3.1. Patients and Stent Graft Design

During the study period, 596 patients underwent a FEVAR procedure for a juxta- or pararenal aneurysm in our centre. Of these patients, 35 (6%) had a PAP/PAA after previous OSR and were included in the study. The demographic characteristics and comorbidities of the patients are shown in Table 1.

Median interval from the primary OSR to the FEVAR procedure was 136 months (range 47–261). Twenty (57%) patients had been previously treated with an aorto-aortic tube graft, seven (20%) with an aorto-biiliac graft, four (11%) with an aorto-bifemoral graft, two (6%) with an aorto-monoiliac graft and two (6%) with an aorto-ilio-femoral graft. The indication for treatment was a PAP in 18 (51%) patients and a PAA in 17 (49%) patients. The mean maximal diameter of the aneurysm was 57 ± 6 mm.

### 3.2. Stent Graft Design and Procedure

**Figure 1:**
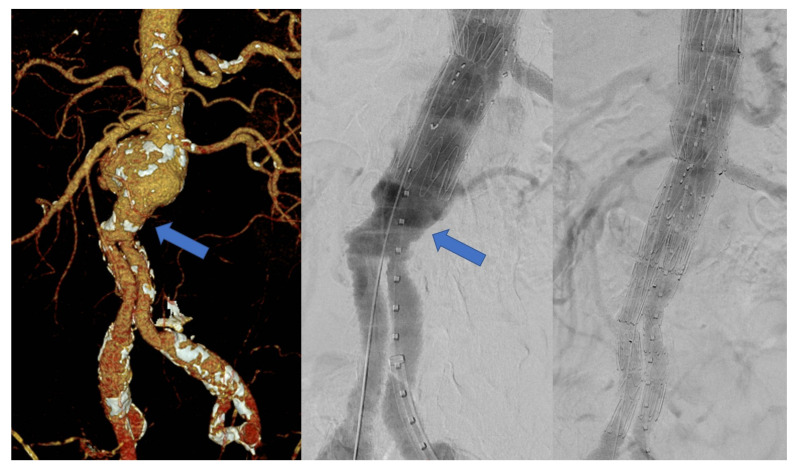
Para-anastomotic aneurysm in a patient with a short body in the previous surgical graft (arrow) and control angiography after treatment with a fenestrated stent graft and a bifurcated device with an inverted limb.

Twenty-nine (83%) patients were treated with a fenestrated tube graft followed by a bifurcated stent graft, five (14%) patients with a fenestrated cuff and one (3%) patient with a fenestrated tube graft followed by an aorto-uniiliac device. This patient had a previous aorto-uniiliac surgical graft after a ruptured AAA. Eight (28%) of the patients that received a bifurcated stent graft were treated with a device including an inverted contralateral limb due to the short working length in the body of the previous surgical graft (Figure 1).

The configuration of the device included fenestrations for the renal arteries only and a scallop for the superior mesenteric artery (SMA) in 11 (31%) cases, fenestrations for the renal arteries and the SMA and a scallop for the celiac artery (CA) in 20 (57%) cases and fenestrations for the renal arteries, SMA and CA in 4 (11%) patients.

Mean operative time was 177 ± 47 min with a mean fluoroscopy time of 46 ± 15 min. The mean iodinated contrast volume used was 152 ± 44 mL.

### 3.3. Technical Success and Early Outcomes

Technical success was achieved in 33 (94%) patients. There were two (6%) technical failures. In one patient with preoperative high-grade stenosis of the right renal artery, it was impossible to catheterize and stent that artery. He had an intraoperative occlusion but presented no endoleak from this fenestration during follow-up. The second technical failure was due to a crushed left renal artery stent as a result of the competition of wires and sheaths in a patient with a very narrow anastomosis from the previous surgical graft. He also did not present any endoleak during follow-up.

There was one (3%) death within the first 30 postoperative days. The patient died two days after the procedure due to complications following postoperative bleeding from one renal artery.

There were no early reinterventions and no cases of spinal cord ischaemia. Five (14%) patients presented with postoperative complications: three patients had a transitory deterioration of renal function without need for haemodialysis. One patient had postoperative pneumonia, and the fifth patient suffered a non-ST elevation myocardial infarction on the second postoperative day. The coronary angiography showed no relevant coronary artery stenosis, and the event was attributed to vasospasm.

The median hospital stay was seven days (range, 5–17 days) and four (11%) patients were admitted to the intensive care unit for postoperative surveillance due to extensive comorbidity.

### 3.4. Late Outcomes

**Figure 2:**
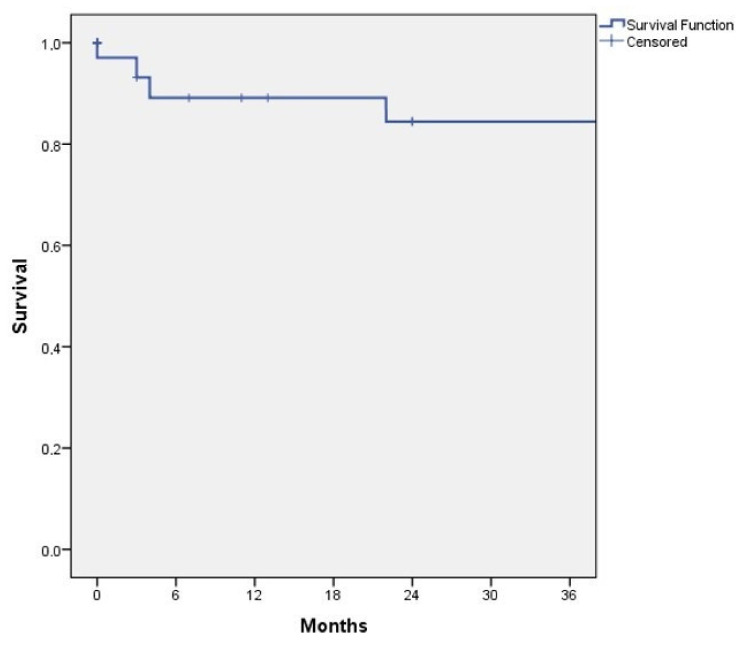
Kaplan–Meyer analysis of patient survival during follow-up.

The median follow-up was 33 months (range 3–121). Estimated overall survival at 12, 24 and 36 months was 89.1% ± 6%, 84.4% ± 7.3% and 84.4% ± 7.3%, respectively. Figure 2 shows the cumulative survival curve estimated by Kaplan–Meier analysis. There was no aneurysm-related mortality in any patient during follow-up.

**Figure 3:**
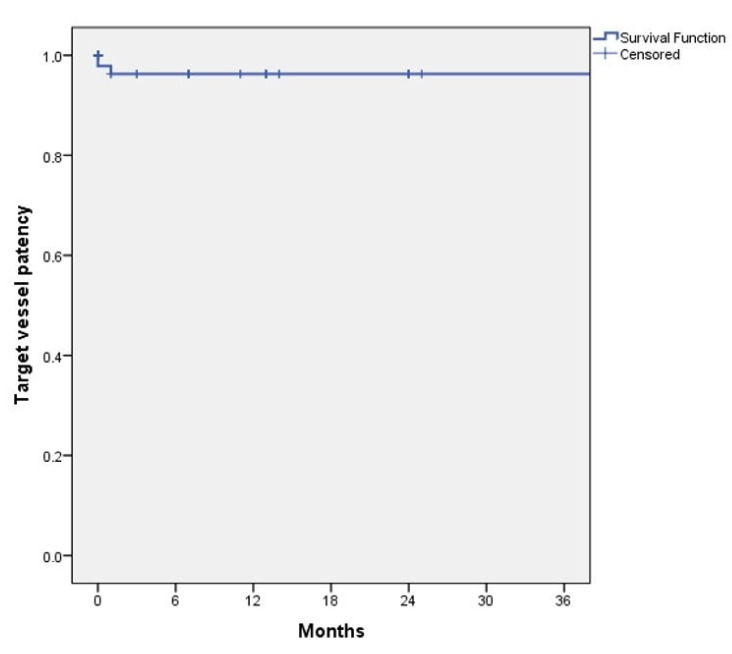
Kaplan–Meyer analysis of target vessel primary patency during follow-up.

One (3%) patient presented with an occlusion of the right renal artery in the first postoperative control. The occlusion was attributed to a small dissection of the vessel that was present in the completion angiography. The patient maintained a stable renal function with a creatinine of 1.7 mg/dL. No other target vessels occluded during follow-up. There was no graft limb occlusion, nor stent graft migration. Figure 3 shows the target vessel’s primary patency estimated by Kaplan–Meier analysis.

**Figure 4:**
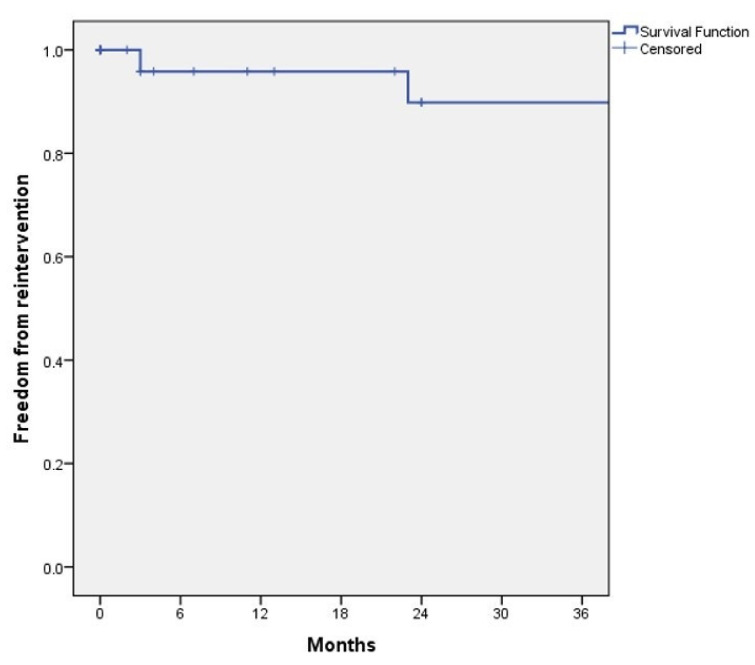
Kaplan–Meyer analysis of freedom from reintervention during follow-up.

Three (9%) patients underwent late reinterventions. The first patient had an extension of the right graft limb for a type Ib endoleak. The second patient had a relining of an iliac graft limb due to kinking of the stent graft. The third patient underwent a reintervention with relining of the left renal artery with a covered stent due to dislocation and relining of the SMA with a self-expandable bare stent due to distal stenosis. Estimated freedom from reintervention at 12, 24 and 36 months was 95.8% ± 4.1%, 89.8% ± 6.9% and 89.8% ± 6.9%, respectively (Figure 4).

## 4. Discussion

Open surgical repair is considered a durable option for the treatment of infrarenal aneurysms. However, the incidence of PAP/PAAs during follow-up reported in the literature ranges from 2% to 9% [17,18,19,20] and this number probably underestimates the real incidence of this pathology due to the inconsistent long-term follow-up of these patients [21]. The median interval from the primary OSR to the FEVAR procedure in our series was 136 months. This shows that PAP/PAAs are frequent even after 10 years of the primary surgery, which highlights the importance of long-term follow-up in patients with OSR too, in order to detect and treat these complications in due time. The current guidelines for the management of AAAs from the European Society for Vascular Surgery recommend follow-up imaging with duplex ultrasound or CTA at 5-year intervals for all patients undergoing OSR for an infrarenal AAA [22].

In earlier times, open surgical reintervention was the only option available for the treatment of this disease and a high percentage of the patients in which a PAP/PAA was discovered were not offered treatment due to the technical challenges and high mortality/morbidity associated with the procedure [6,7,8,19]. The scarring produced by the previous operation, involvement of reno-visceral vessels with need for suprarenal/supravisceral clamping and the advanced age of the patients with possible progression of cardiac and pulmonary comorbidity has led to a shift towards endovascular options for this pathology.

Standard EVAR was frequently used to treat these cases in the beginning, but some studies showed a high incidence of type I endoleaks in patients with endovascular aortic tube grafts for proximal anastomotic aneurysms, highlighting the importance of suitable anatomy to achieve a durable endovascular repair in this disease [23]. This suitable anatomy is only available in a minority of patients, as the absence of a healthy infrarenal segment of the aorta proximal to the PAP/PAA precludes the use of standard EVAR in around 80% of the cases with involvement of the proximal anastomosis [24].

In the last years, FEVAR has emerged as a less invasive alternative for the treatment of PAP/PAAs and it has since become the first-line treatment option for this disease in some centres [25,26]. The present study suggests that FEVAR is a safe and effective alternative for these patients offering a high rate of technical success and good mid-term results with low morbidity and mortality and high target-vessel patency during follow-up. The previous experience with this technique reported by our group [15] was included in a systematic review published in 2020 by Spanos et al. that analysed the results of eighteen studies with a total of 433 patients treated endovascularly for a PAP/PAA [27]. In this review, 23% of the patients were treated with FEVAR showing high technical success rates and primary patency of the renovisceral vessels of 97.4% during follow-up.

Some studies have compared the results of fenestrated and branched endovascular aortic repair (F/B-EVAR) between patients with PAP/PAAs and those treated for a primary aneurysm. Gallitto et al. analysed the outcomes of F/B-EVAR in 32 patients with previous OSR and 30 patients treated for a primary aneurysm finding no differences in terms of technical success, mortality or target vessel patency between groups [26]. These promising outcomes were later confirmed in a larger series published by the author that included 108 patients treated with F/B-EVAR for a PAP/PAA in two different centres in Italy and France [28]. Another recent study by D’Oria et al. also found similar outcomes in a series including 58 patients with prior infrarenal repair (EVAR = 33, OSR = 25) and 164 patients with primary aneurysms. The results suggested that F/B-EVAR is equally safe and feasible for the treatment of patients with prior infrarenal repair as compared with those undergoing treatment for native aneurysms. An increased rate of target vessel-related endoleaks was found in patients with a prior repair in this study. However, this increase did not correlate with higher rates of reinterventions or lower primary patency at 5-year follow-up [5].

Despite the good rates of technical success reported in all these series, FEVAR after open repair is associated with additional technical challenges during the planning and execution of this procedure. The most common configuration of fenestrated devices nowadays includes a proximal fenestrated tube followed by a distal bifurcated stent graft. This configuration allows for easy rotation and repositioning of the proximal component to facilitate target vessel catheterization and prevents migration of the stent graft, stabilising the repair. However, the standard use of a short surgical graft body with longer limbs for patients with aorto-biiliac or aorto-bifemoral reconstructions complicates planning due to the short working length proximal to the surgical graft bifurcation. While treatment with fenestrated cuffs has proven to be an option in patients with these anatomical characteristics, this configuration has been associated with higher rates of distal reinterventions compared to complete relining of the original repair [29]. For this reason, we prefer the use of bifurcated stent grafts with an inverted contralateral limb (Figure 1) in those cases with enough working length to accommodate this device proximal to the bifurcation.

The presence of a surgical graft also involves additional intraoperative challenges. A narrow body/limb limits the manoeuvrability and repositioning of the device, which can make the catheterisation of target vessels cumbersome. The use of single/double diameter reducing ties, as offered in the Zenith fenestrated platform, is highly recommended in these cases in order to avoid excessive frictional forces with the surgical graft and facilitate the rotation of the graft. Competition of wires, catheters and sheaths in patients with a narrow proximal anastomosis can also incur additional complications: In our series, it caused the crushing and occlusion of a renal artery stent with subsequent loss of the kidney in one patient.

This study has limitations in view of the retrospective design and the small number of patients. As reflected by the nature of the disease, no prospective studies are available to date and the literature reporting the use of FEVAR in patients with PAP/PAA is limited to small single-centre series or multicentre registries. Finally, it must be acknowledged that our centre has a large experience in complex aneurysm endovascular repair and therefore outcomes may not be generalisable to centres with lower volumes.

In conclusion, FEVAR is a safe and effective alternative for the endovascular treatment of PAP/PAAs after OSR showing high technical success, low mortality and morbidity, and good mid-term outcomes.

## Figures and Tables

**Table 1:** **Baseline and comorbidity characteristics.** Data expressed in absolute numbers (percentage), except where indicated: mean (Standard Deviation). COPD: chronic obstructive pulmonary disease.

N = 35	
Mean age (SD)	72.9 (7)
Male sex	34 (97%)
Hypertension	23 (66%)
Hypercholesterolaemia	14 (20%)
Diabetes mellitus	4 (11%)
Smoking (current or past)	19 (54%)
Coronary artery disease	8 (23%)
COPD	17 (49%)
Serum creatinine > 100 µmol/L	16 (46%)
ASA Physical Status ≥ III	24 (69%)
